# Differential electrophysiological properties of D1 and D2 spiny projection neurons in the mouse nucleus accumbens core

**DOI:** 10.14814/phy2.13784

**Published:** 2018-07-01

**Authors:** Nour Al‐muhtasib, Patrick A. Forcelli, Stefano Vicini

**Affiliations:** ^1^ Department of Pharmacology & Physiology Georgetown University Medical Center Washington District of Columbia; ^2^ Interdisciplinary Program in Neuroscience Georgetown University Medical Center Washington District of Columbia

**Keywords:** Nucleus accumbens, ventral striatum

## Abstract

The striatum consists of the dorsal (caudate/putamen) and the ventral (nucleus accumbens) regions. The nucleus accumbens is further divided into a core and shell. Both the dorsal and ventral striatum contain populations of spiny projection neurons, which make up 95% of the neurons within the striatum. SPNs are canonically categorized into those that express the D1‐type dopamine receptor (D1 SPNs) and those that express the D2‐type dopamine receptor (D2 SPNs). D1 and D2 SPNs differ with respect to both synaptic inputs and projection targets. In the dorsal striatum, it is well established that these populations of SPNs differ in terms of their electrophysiological and morphological properties. However, there remains a gap in our knowledge of the electrophysiological properties of SPNs in the nucleus accumbens core. To evaluate the differential properties of these SPNs, we performed whole‐cell recordings from D1 and D2 SPNs in BAC transgenic mice in which D1 SPNs fluoresce red and D2 SPNs fluoresce green. The two SPN subtypes did not differ in terms of their time constant, capacitance, resting membrane potential, or tonic current. However, D2 SPNs displayed heightened inhibitory postsynaptic current (IPSC) and miniature excitatory PSC frequency as compared with D1 SPNs. Furthermore, D2 SPNs displayed decreased rheobase, increased excitability as measured by firing rates to depolarizing current injections, increased inward rectification, increased input resistance, and decreased dendritic complexity compared to D1 SPNs. Our results demonstrate a dichotomy in the electrophysiological properties of D1 and D2 SPNs in the nucleus accumbens core, which contributes to our knowledge of ventral striatal circuitry.

## Introduction

The dorsal (caudate/putamen) and ventral striatum (nucleus accumbens, NuAcc) contain populations of GABAergic spiny projection neurons (SPNs) which make up 95% of the neuronal population. The remaining 5% of neurons consist of GABAergic and cholinergic interneurons (Kawaguchi et al. 1995; Gerfen and Wilson 1996). The SPNs consist of two subtypes, D1‐type dopamine receptor containing SPNs (D1 SPNs) and D2‐type dopamine receptor containing SPNs (D2 SPNs), and are the main output neurons of the striatum. The soma of spiny projections neurons are small to medium in size (diameter: 9–15 *μ*m) with spiny dendrites (Meredith et al. 1992). In both the dorsal and ventral striatum, SPNs exhibit resting membrane potentials close to the potassium equilibrium potential, partly due to inwardly rectifying potassium channels (*I*_Kir_) (Uchimura et al. 1989; Nisenbaum and Wilson 1995).

The electrophysiological properties of D1 and D2 SPNs in the dorsal striatum have been characterized extensively (Gertler et al. 2008). In the dorsal striatum, D1 SPNs display a lower input resistance, membrane resistance, and membrane time constant in comparison with D2 SPNs (Gertler et al. 2008). Despite the greater rheobase in D1 SPNs, spike threshold did not differ between D1 and D2 SPNs (Gertler et al. 2008). D1 SPNs display greater conductance through I_Kir_, which plays a crucial role in the maintenance of SPN down state (Nisenbaum and Wilson 1995) and, in part, is reflected in a hyperpolarized resting membrane potential compared with D2 SPNs (Gertler et al. 2008). In addition, dorsal striatal D1 SPNs displayed a higher whole‐cell capacitance along with increased dendritic area and number of primary dendrites (Gertler et al. 2008). Further differences include larger GABA_A_ receptor‐mediated tonic currents in D2 SPNs than in D1 SPNs in younger animals (postnatal day 16–25, P16–P25 mice) (Ade et al. 2008; Santhakumar et al. 2010). In older animals (>P30 mice), tonic currents increased in D1 SPNs but decreased in D2 SPNs (Santhakumar et al. 2010).

As compared with the dorsal striatum, less is known regarding the differences between D1 and D2 SPNs in the NuAcc. Morphologically, SPNs of the NuAcc core and NuAcc shell (Meredith et al. 1992) differ, with neurons in the core displaying higher spine densities, a greater degree of dendritic branching, and increased synaptic terminals. Consistent with this, core SPNs have a higher surface area than shell SPNs (Meredith et al. 1992). NuAcc SPNs differ from dorsal striatal SPNs with respect to dopamine receptor expression: a larger subset of neurons in the NAcc expresses both D1‐ and D2‐type dopamine receptors. This degree of receptor colocalization is higher in the NuAcc core (21%) than in the NuAcc shell (13%) (Kupchik et al. 2015). The differences between D1 and D2 SPNs also extend to functional properties; for example, D2 SPNs are more excitable and display a larger ratio of NMDA receptor mediated excitatory postsynaptic currents (Gokce et al. 2016). While these properties of SPNs in the ventral striatum have been investigated, the differences between the two SPN subtypes in terms of their passive properties and synaptic input remain unexplored. Taking into consideration the ventral striatum's vital role in reward and motor function, these data are essential to better to understand the electrophysiological profile of ventral striatal SPN subtypes. To address this gap, we performed whole‐cell recordings from D1 and D2 SPNs in acute slices made from mice in genetically identified D1 and D2 SPNs and compared the intrinsic cell properties and synaptic transmission in these two populations.

## Methods

### Animals and genotyping

Bacterial artificial chromosome (BAC) D2 enhanced green fluorescent protein (EGFP) and BAC D1 tdTomato mice were crossed to obtain a mouse that expresses both D2‐EGFP and D1‐tdTomato (Gong et al. 2003; Shuen et al. 2008). All mice were maintained to the C57BL/6 background. At postnatal days 0–1 (P0–P1), mice were light genotyped for red fluorescence using a dual fluorescent protein flashlight (NightSea, Lexington, MA). At P7, animal genotype for GFP was assessed by tail biopsy conducted by Transnetyx, Inc. (Cordova, TN, USA). All mice were group‐housed in barrier cages in rooms with a 12‐h:12‐h light/dark cycle. They were permitted free access to food and water. Both male and female mice were used for all studies and were combined for all analyses, as they did not differ statistically on the parameters analyzed by sex. The procedures performed in this manuscript were performed in accordance with and approval by Georgetown University Animal Care and Use Committee. The number of animals and cells were as follows: passive properties (D1 SPNs = 19 cells from 14 animals, D2 SPNs = 17 cells from 13 animals), active properties (D1 SPNs = 8 cells from 7 animals, D2 SPNs = 10 cells from 6 animals), inward rectification (D1 SPNs = 16 cells from 9 animals, D2 SPNs = 10 cells from 9 animals), TTX‐sensitive tonic current (D1 SPNs = 13 cells from 10 animals, D2 SPNs = 13 cells from 10 animals), BMR‐sensitive tonic current (D1 SPNs = 12 cells from 9 animals, D2 SPNs = 11 cells from 7 animals), sIPSCs (D1 SPNs = 14 cells from 10 animals, D2 SPNs = 14 cells from 11 animals), mIPSCs (D1 SPNs = 9 cells from 8 animals, D2 SPNs = 11 cells from 7 animals), mEPSCs (D1 SPNs = 9 cells, D2 SPNs = 9 cells), dendritic architecture (D1 SPNs = 13 cells from 10 animals, D2 SPNs = 10 cells from 6 animals).

### Brain slice preparation

Slices were prepared from P17–23 male and female mice to compare with previous work in our lab (Ade et al. 2008; Janssen et al. 2009). Mice were killed by rapid decapitation in agreement with the guidelines of the American Veterinary Medical Association Panel on Euthanasia and the Georgetown University Animal Care and Use Committee. The whole brain was removed and placed in an ice‐cold cutting solution containing (in mmol/L): NaCl (87.3), KCl (2.7), CaCl_2_ (0.5), MgSO_4_ (nonhydrate) (6.6), NaH_2_PO_4_ (1.4), NaHCO_3_ (26.0), dextrose (25.0), sucrose (75.1) (all from Sigma, St. Louis, MO, USA). A Vibratome 3000 Plus Sectioning System (Vibratome, St. Louis, MO, USA) was used to prepare 250‐*μ*m thick striatal coronal slices. The slices were incubated in artificial cerebrospinal fluid (aCSF) containing (in mmol/L) NaCl (123.9), KCl (4.5), Na_2_HPO_4_ (1.2), NaHCO_3_ (26.0), CaCl_2_ (2.0), MgCl_2_ (1.0), and dextrose (10.0) at 305 mOsm at 32°C for 30 min. The slices were then incubated for an additional 30 min in the same solution, which was also used as the extracellular recording solution at room temperature. All solutions were continuously bubbled with 95% O_2_/5% CO_2_ to maintain a pH of 7.4.

### Whole‐cell recordings

Slices were visualized using an upright microscope (E600FN, Nikon, Tokyo, Japan) equipped with Nomarski optics and a 60X water immersion objective with a long working distance (2 mm) and high numerical aperture (1.0). Recording electrodes with a resistance of 3–5 MΩ were prepared from borosilicate glass capillaries (Wiretrol II; Drummond, Broomall, PA, USA).

A KCl‐based internal solution containing (in mmol/L) KCl (145.0), HEPES (10.0), ATP‐Mg (5.0), GTP‐Na (0.2), EGTA (5.0) and adjusted to pH 7.2 with KOH was used for all recordings. A KCl‐based internal solution was used to visualize GABAergic currents at −70 mV. Voltage‐clamp recordings were achieved using the whole‐cell configuration method at a holding voltage of −70 mV using the MultiClamp 700B amplifier (Molecular Devices, San Jose, CA, USA). All recordings were performed at room temperature, 22–24°C. Recordings were performed from D1 and D2 SPNs in the ventral striatum, in the area directly surrounding the anterior commissure (NuAcc core).

Recordings were performed from neurons that displayed only red (D1+) or green (D2+) fluorescent reporters; the small population of double‐labeled neurons putatively expressing both D1‐ and D2‐type dopamine receptors (Kupchik et al. 2015) were excluded from recordings. Responses to increasing hyperpolarizing and depolarizing current injections from rest (10 pA steps) were obtained to assess passive properties as well as action potential number and firing pattern. Access resistance was monitored periodically during voltage‐clamp experiments, and recordings with a >20% change were discarded. Current clamp recordings were intercalated between voltage‐clamp experiments. Recordings were filtered at 2 kHz with a low‐pass Bessel filter and digitized at 20 kHz using a personal computer equipped with Digidata 1440 data acquisition board and pCLAMP10 software (both from Molecular Devices).

### Drugs

Working solutions of tetrodotoxin (TTX, 1 *μ*mol/L) and bicuculline methobromide (BMR, 25 *μ*mol/L, both from Sigma) were prepared in aCSF and locally applied to the slice via Y tube (Hevers and Lüddens 1998). Prior to drug application, the whole‐cell currents were acquired for 5 min to obtain spontaneous inhibitory postsynaptic currents (sIPSCs), at which time TTX was applied to study miniature IPSCs (mIPSCs); lastly BMR was applied to evaluate GABA_A_ mediated tonic current and isolate glutamatergic currents (mEPSCs). NBQX was not used for the measurement of IPSCs as to not disturb the network activity (Brickley and Mody 2012). The rapid decay kinetics of AMPA‐mediated EPSCs allowed us to exclude them from IPSC analysis (Ortinski et al. 2006; Janssen et al. 2011; Forcelli et al. 2012; Al‐Muhtasib et al. 2018). In acute corticostriatal slices, sEPSC and mEPSC frequency are equivalent, and thus, we only recorded mEPSCs (Forcelli et al. 2012).

### Measurement of electrophysiological properties

Our primary goal was to assess GABAergic synaptic input to D1 and D2 SPNs, and the experiments were designed to facilitate these measurements. However, to maximize the data collected, we also explored other parameters of interest.

IPSCs were measured using ClampFit template search and visually confirmed (Janssen et al. 2011; Forcelli et al. 2012). Peak amplitude, rise time, decay time, and inter‐event interval were obtained from the ClampFit results. Frequency of IPSCs was measured directly as the number of events divided by the length of the recording. Tonic current was analyzed using an all‐points histogram that measured the shift in mean holding current 5 sec before and during TTX or BMR application (5 sec). The BMR‐sensitive tonic current was calculated from the shift in baseline, in addition to the TTX‐sensitive tonic current. Contamination by synaptic events was considered and is negligible as demonstrated by other groups (Nusser and Mody 2002).

Resting membrane potential was measured at *I* = 0, and it was not corrected for liquid junction potential (typically ~3 mV with a KCl solution). Passive properties were measured from the voltage response to hyperpolarizing (−10 pA) current injections. Input resistance was calculated as the slope of the linear portion of the voltage–current curve. The time constant was measured from the decay of the voltage response to hyperpolarizing current injections. Membrane capacitance was measured indirectly from the input resistance and time constant. Action potential firing rate was measuring manually from depolarizing current injections. Rheobase was considered to be the first depolarizing current injection to induce an action potential. A neuron with more inward rectification will have a decreased voltage response to large hyperpolarizing current injections. As such, inward rectification was measured from the ratio (Kir ratio) of the difference in voltage response between the two largest hyperpolarizing current injections (−110 pA and −120 pA) and the difference in resting membrane potential and voltage response to the first −10 pA current injection from rest. The data points used for measurements were selected from linear portions of the current voltage relationship.

### Morphological reconstruction

Whole‐cell recordings were obtained using the same internal solution detailed above with the addition of 0.5% biocytin. Neurons were then injected with up to 35 steps of hyperpolarizing and depolarizing current injections in current clamp mode (100 msec, 20 pA). After approximately 15 min of recording, an outside‐out seal was obtained, and slices were allowed to rest for another 45 min before fixation. Slices were then fixed with 4% sucrose/4% paraformaldehyde in 0.1 mol/L phosphate buffered saline (PBS) at room temperature for 2 h. The slices were subsequently washed with 0.5% triton‐X in 1X PBS for at least 30 min at room temperature (RT). Slices were then incubated in avidin‐fluorescein (2.5 *μ*L/mL) for 2 h at RT. Afterward slices were washed overnight at 4°C and then mounted with Vectashield, H‐1000 mounting medium (Vector Laboratories, Burlingame, CA) to be imaged.

Imaging of slices was performed using a ThorLabs resonance laser scanning confocal microscope with 488‐ and 547‐nm argon laser on a Nikon Eclipse FN1 upright microscope with a 60× water immersion lens (1.0 N.A.), a 40× lens (0.9 N.A.), or a 20× lens (0.5 N.A.).

Biocytin injected cells were traced using the Fiji NeuronJ plugin, and dendritic arborization was analyzed using the Sholl analysis plugin (Ferreira et al. 2004). Primary branches originated directly from the soma, secondary branches originated from primary branches, and branching from then on was considered to be tertiary branching. Dendritic length was measured using the NeuronJ plugin.

### Experimental design and statistical analysis

Bar graphs of frequency, peak amplitude, rise time, and decay time of postsynaptic currents display mean values and standard error of the mean. Statistical analyses were conducted using GraphPad Prism. Outliers were determined using the ROUT test (*Q* = 1%) and were excluded from all datasets. Excluded data are as follows: passive properties (D1 SPNs = 1, D2 SPNs = 0), active properties (D1 SPNs = 0, D2 SPNs = 3), inward rectification (D1 SPNs = 2, D2 SPNs = 5), TTX‐sensitive tonic current (D1 SPNs = 0, D2 SPNs = 0), BMR‐sensitive tonic current (D1 SPNs = 0, D2 SPNs = 0), sIPSCs (D1 SPNs = 1, D2 SPNs = 3), mIPSCs (D1 SPNs = 4, D2 SPNs = 2), mEPSCs (D1 SPNs = 3, D2 SPNs = 2), and dendritic architecture (D1 SPNs = 2, D2 SPNs = 4).

Electrophysiological and morphological reconstruction data were analyzed as a function of cell type. The data were tested for normality and the appropriate parametric or nonparametric tests were used. For IPSC parameters and passive properties, statistical significance was assessed using the nonparametric Mann–Whitney test. Neuronal firing pattern was analyzed by two‐way ANOVA with cell type as a between subject factor and current intensity as a within subject factor. Morphological analyses were conducted using a two‐way ANOVA for the Sholl analysis, Kruskal–Wallis for the length, and individual branch count parameters. In all cases, *P* values less than 0.05 were considered to be statistically significant.

## Results

### Passive and active properties

**Figure 1:**
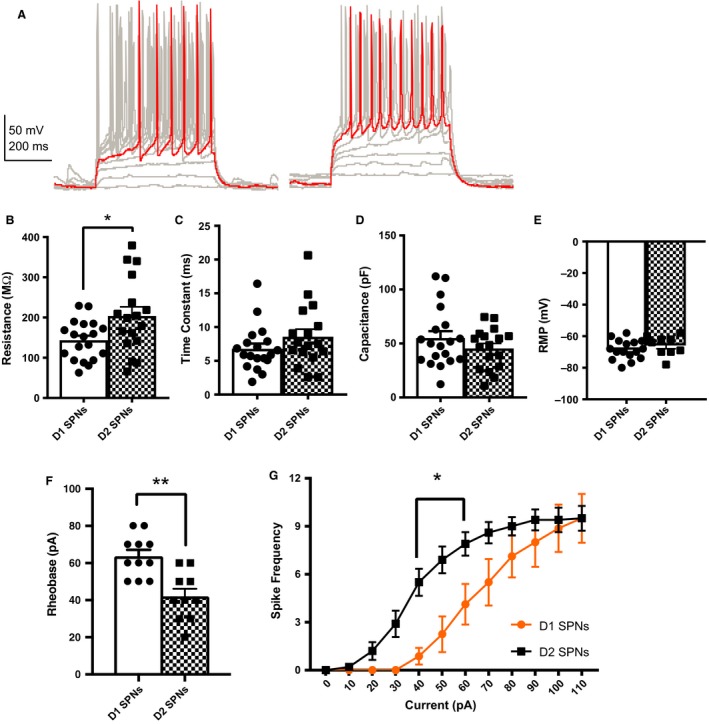
D2 SPNs display increased excitability compared with D1 SPNs. (A) Representative traces from current clamp recordings of D1 SPNs (left) and D2 SPNs (right). (B) Input resistance was increased in D2 SPNs (204 ± 22.6 MΩ) compared with D1 SPNs (144 ± 11.2 MΩ, *P* = 0.0449). (C) Time constant (D1 SPNs = 7 ± 0.8 msec, D2 SPNs = 9 ± 1.1 msec, *P* = 0.1726), and (D) Capacitance (D1 SPNs = 55 ± 6.4 pF, D2 SPNs = 45 ± 4.6 pF,* P* = 0.5308) did not differ between D1 and D2 SPNs in the nucleus accumbens core. *N* = D1 SPNs (19 cells from 14 animals), D2 SPNs (17 cells from 13 animals). (E) The resting membrane potential (RMP) of D1 SPNs (−69 ± 1.5 mV) and D2 SPNs (−66 ± 2.1 mV,* P* = 0.2801) did not differ. (F) The rheobase of D2 SPNs (42 ± 4.2 pA) was significantly lower than that of D1 SPNs (64 ± 3.4 pA,* P* = 0.0013). (G) D2 SPNs displayed increased excitability compared with D1 SPNs. Increasing amplitude of depolarizing current steps led to a significant increase in action potential firing rate in D1 and D2 SPNs (*F*_11,176_ = 80.55, *P* < 0.0001). There was a main effect of cell type (*F*_1,16_ = 4.775, *P* = 0.0441) and a current step by cell type interaction (*F*_11,176_ = 4.502, *P* < 0.0001). The source of this difference was between the 40 pA (*P* = 0.0023) and 60 pA (*P* = 0.0257) steps. *N* = D1 SPNs (8 cells from 7 animals), D2 SPNs (10 cells from 6 animals). Mann–Whitney test was used for input resistance, time constant, capacitance, RMP, and rheobase. Two‐way ANOVA with multiple comparisons for the input–output curve.

Input resistance was increased in D2 SPNs (204 ± 22.6 MΩ) compared with D1 SPNs (144 ± 11.2 MΩ, *P* = 0.0449, Fig. 1B). However, the SPN subtypes did not differ in terms of their time constant (D1 SPNs = 7 ± 0.8 msec, D2 SPNs = 9 ± 1.1 msec, *P* = 0.1726, Fig. 1C) and whole‐cell capacitance (D1 SPNs = 55 ± 6.3 pF, D2 SPNs = 45 ± 4.6 pF, *P* = 0.5308, Fig. 1D). D1 SPNs (−69 ± 1.5 mV) and D2 SPNs (−66 ± 2.1 mV) displayed hyperpolarized resting membrane potentials typical of SPNs but did not differ from each other (*P* = 0.2801, Fig. 1E).

Although D1 SPNs and D2 SPNs of the NuAcc core did not differ in all of the above membrane properties, there were differences in a subset of their active properties. Consistent with prior findings, NAcc core D2 SPNs displayed decreased rheobase and increased excitability compared with D1 SPNs (Grueter et al. 2010). The rheobase of D2 SPNs (42 ± 4.2 pA) was significantly lower than that of D1 SPNs (64 ± 3.4 pA, *P* = 0.0013, Fig. 1A and F). Increasing amplitude of depolarizing current steps led to a significant increase in action potential firing rate in both D1 and D2 SPNs (Fig. 1G, *F*_11,176_ = 80.55, *P* < 0.0001). There was a main effect of cell type (*F*_1,16_ = 4.775, *P* = 0.0441) and a current step by cell type interaction (*F*_11,176_ = 4.502, *P* < 0.0001). The source of this difference was the firing rates between the 40 pA (*P* = 0.0023) and 60 pA (*P* = 0.0257) steps.

**Figure 2:**
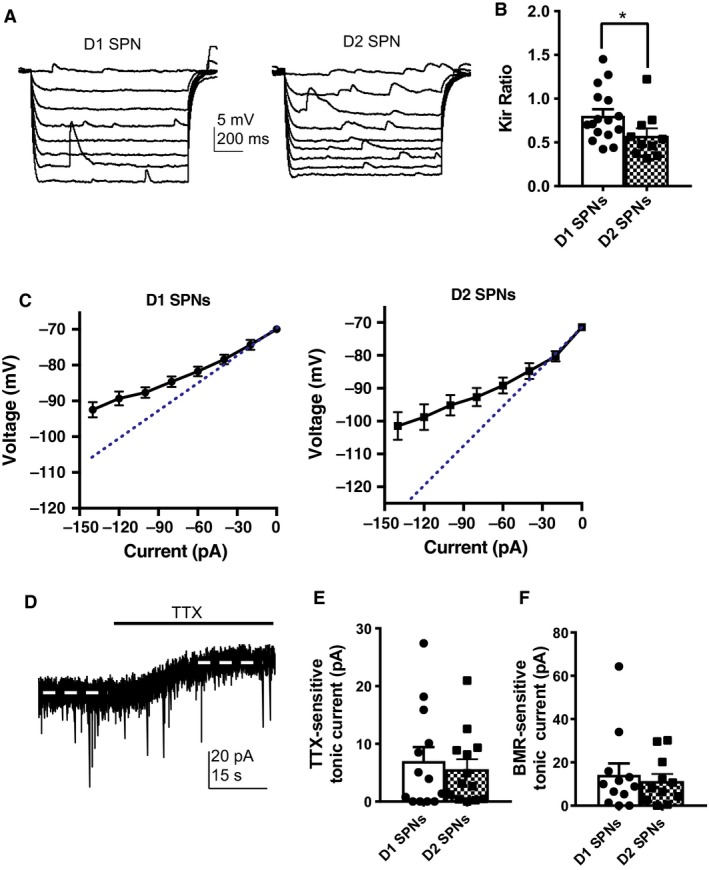
Increased inward rectification in D2 SPNs. (A) Responses of D1 SPNs (left) and D2 SPNs (right) to 20 pA hyperpolarizing current pulses (500 msec duration). (B) D2 SPNs (0.58 ± 0.08) displayed a decreased Kir ratio compared with D1 SPNs (0.80 ± 0.07, *P* = 0.0309). *N* = D1 SPNs (16 cells from 9 animals), D2 SPNs (10 cells from 9 animals). (C) Current–Voltage plots for D1 and D2 SPNs. Dotted line represents what the slope of the plot would have been without inward rectification. (D) Example trace of TTX‐sensitive tonic current from a responding cell. (E) TTX application revealed the presence of a tonic current in both D1 SPNs (7.0 ± 2.4 pA) and D2 SPNs (5.6 ± 1.7 pA,* P* = 0.9597). (F) BMR application revealed tonic current in both D1 SPNs (14.23 ± 5.3 pA) and D2 SPNs (11.4 ± 3.3 pA,* P* = 0.8800). *N* = TTX‐sensitive tonic current (D1 SPNs = 13 cells from 10 animals, D2 SPNs = 13 cells from 10 animals), BMR‐sensitive tonic current (D1 SPNs = 12 cells from 9 animals, D2 SPNs = 11 cells from 7 animals). Mann–Whitney test.

SPNs display hyperpolarized resting membrane potentials in part due to the activation of I_Kir_ (Nisenbaum and Wilson 1995). We, therefore, assessed the extent of inward rectification via a Kir ratio as described in the methods. There data revealed a significantly lower Kir ratio in D2 SPNs (0.58 ± 0.08) compared with D1 SPNs (0.80 ± 0.07, *P* = 0.0309), indicating increased rectification in D2 SPNs (Fig. 2A–C).

Previous work has reported a larger tonic current in D2 SPNs of the dorsal striatum compared with D1 SPNs (Ade et al. 2008). TTX application revealed the presence of a tonic current in both D1 SPNs (7 ± 2.4 pA) and D2 SPNs (5 ± 1.7 pA, *P* = 0.9597, Fig. 2D and E). Similarly, BMR application revealed tonic current in both D1 SPNs (14 ± 5.3 pA) and D2 SPNs (11 ± 3.3 pA, *P* = 0.8800, Fig. 2F).

### Synaptic transmission

**Figure 3:**
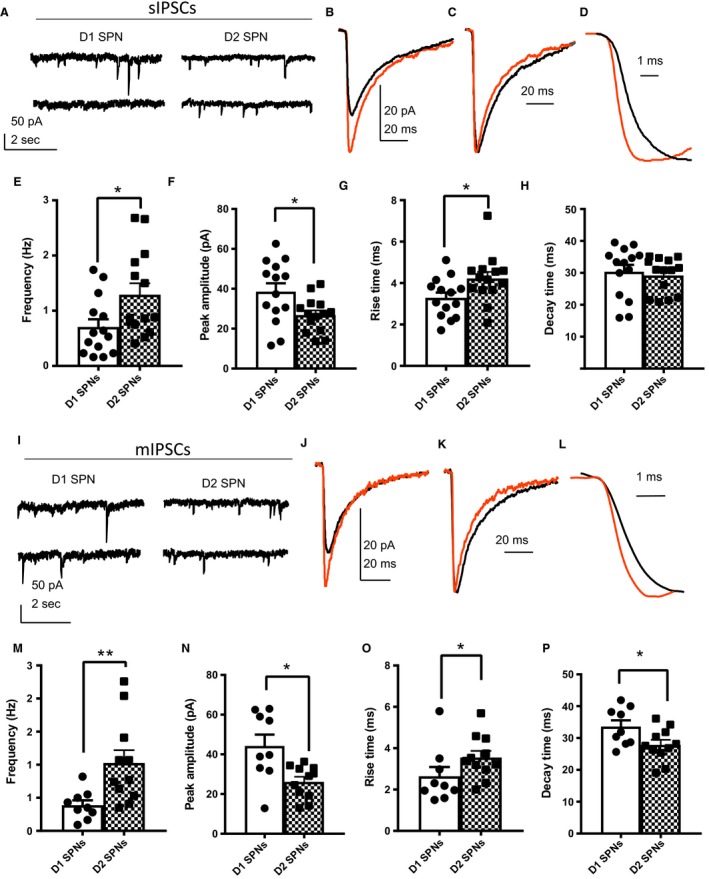
Increased sIPSC and mIPSC frequency in D2 SPNs compared with D1 SPNs. sIPSCs (A–H), mIPSCs (I–P). (A) Representative traces of whole‐cell voltage‐clamp recordings (sIPSCs) from D1 SPNs (left) and D2 SPNs (right). (B) Averaged waveforms of sIPSCs from D1 SPNs (orange) and D2 SPNs (black). (C) Normalized waveforms from Figure B. (D) Figure C magnified to display the differences in rise time. (E) D2 SPNs (1.3 ± 0.21 Hz) displayed increased sIPSC frequency compared with D1 SPNs (0.71 ± 0.14 Hz, *P* = 0.0300). (F) Peak amplitude of D1 SPNs (39 ± 4.2 pA) was significantly larger than that of D2 SPNs (27 ± 2.3 pA,* P* = 0.0395). (G) Rise times were significantly longer in D2 SPNs (4.2 ± 0.31 msec) compared with D1 SPNs (3.3 ± 0.26 msec, *P* = 0.0282). (H) Decay times did not differ between D1 (30 ± 2.2 msec) and D2 SPNs (29 ± 1.5 msec, *P* = 0.4668). *N* = D1 SPNs (14 cells from 10 animals), D2 SPNs (14 cells from 11 animals). Mann–Whitney test. (I) Representative traces of whole‐cell voltage‐clamp recordings (mIPSCs) from D1 SPNs (left) and D2 SPNs (right). (J) Averaged waveforms of sIPSCs from D1 SPNs (orange) and D2 SPNs (black). (K) Normalized waveforms from Figure J. (L) Figure K magnified to display the differences in rise time. (M) mIPSC frequency was significantly higher in D2 SPNs (1.0 ± 0.19 Hz) compared with D1 SPNs (0.39 ± 0.07 Hz, *P* = 0.0045). (N) Peak amplitude was significantly smaller in D2 SPNs (26 ± 2.5 pA) compared with D1 SPNs (44 ± 5.8 pA,* P* = 0.0133). (O) Rise times were longer in D2 SPNs (3.5 ± 0.32 msec) compared with D1 SPNs (2.6 ± 0.45 msec, *P* = 0.0381). (P) Decay times were shorter in D2 SPNs (28 ± 1.6 msec) compared with D1 SPNs (33 ± 1.7 msec, *P* = 0.0381). *N* = D1 SPNs (9 cells from 8 animals), D2 SPNs (11 cells from 7 animals). Mann–Whitney test.

In addition to differences in their active properties, D1 and D2 SPNs differed in the extent of inhibitory synaptic transmission they receive (Fig. 3A–H). Spontaneous inhibitory postsynaptic current (sIPSC) frequency was significantly higher in D2 SPNs (1.3 ± 0.21 Hz) compared with D1 SPNs (0.71 ± 0.14 Hz, *P* = 0.0300, Fig. 3E). Peak amplitude was significantly smaller in D2 SPNS (27 ± 2.33 pA) compared with D1 SPNs (39 ± 4.20 pA, *P* = 0.0395**,** Fig. 3F). In addition, rise times were significantly longer in D2 SPNs (4.2 ± 0.31 msec) compared with D1 SPNs (3.3 ± 0.26 msec, *P* = 0.0282, Fig. 3G). Decay time did not differ between D1 (30 ± 2.2 msec) and D2 SPNs (29 ± 1.5 msec, *P* = 0.4274, Fig. 3H).

Action potential independent inhibitory transmission was also assessed via application of the voltage‐gated sodium channel blocker, TTX (Fig. 3I–P). Miniature IPSC (mIPSC) frequency was significantly higher in D2 SPNs (1.0 ± 0.19 Hz) compared with D1 SPNs (0.39 ± 0.07 Hz, *P* = 0.0045, Fig. 3M). Peak amplitude was significantly smaller in D2 SPNs (26 ± 2.5 pA) compared with D1 SPNs (44 ± 5.8 pA, *P* = 0.0133, Fig. 3N). Rise times were longer in D2 SPNs (3.6 ± 0.32 msec) compared with D1 SPNs (2.6 ± 0.45 msec, *P* = 0.0381, Fig. 3O). Surprisingly, decay times were shorter in D2 SPNs (28 ± 1.6 msec) compared with D1 SPNs (33 ± 1.7 msec, *P* = 0.0381, (Fig. 3P).

**Figure 4:**
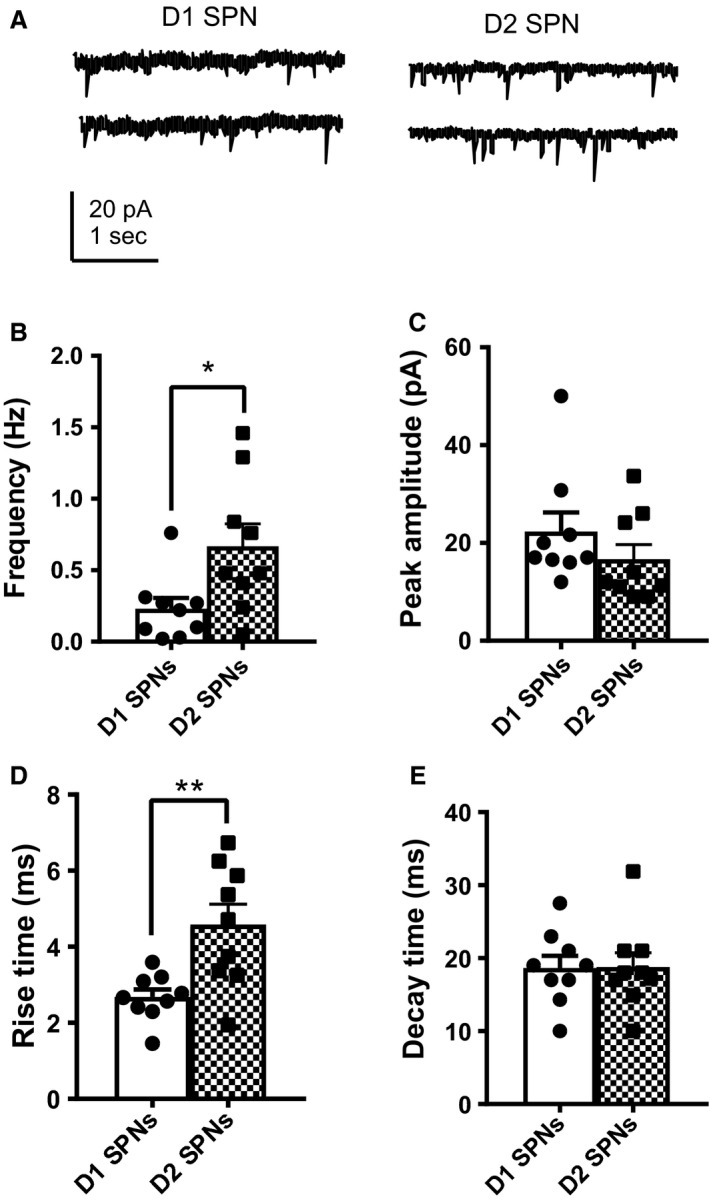
Increased mEPSC frequency in D2 SPNs compared with D1 SPNs. (A) Representative traces of mEPSC whole‐cell voltage‐clamp recordings from D1 SPNs (left) and D2 SPNs (right). (B) mEPSC frequency was significantly higher in D2 SPNs (0.67 ± 0.16 Hz) compared with D1 SPNs (0.23 ± 0.08 Hz, *P* = 0.0198). (C) mEPSC peak amplitude did not differ between D1 (22 ± 3.9 msec) and D2 SPNs (17 ± 3.0 pA,* P* = 0.1411). (D) mEPSC rise times were significantly longer in D2 SPNs (4.6 ± 0.54 msec) compared with D1 SPNs (2.7 ± 0.21 msec, *P* = 0.0056). (E) Decay times did not differ between SPN subtypes. (D1 SPNs = 19 ± 1.7 pA, D2 SPNs = 19 ± 2.0 pA *P* = 0.9524). *N* = D1 SPNs (9 cells), D2 SPNs (9 cells). Mann–Whitney test.

Excitatory transmission was also assessed post‐TTX application. Miniature excitatory PSC (mEPSC) frequency was significantly higher in D2 SPNs (0.67 ± 0.16 Hz) compared with D1 SPNs (0.23 ± 0.08 Hz, *P* = 0.0198, Fig. 4A and B). In addition, mEPSC rise times were significantly longer in D2 SPNs (4.6 ± 0.54 msec) compared with D1 SPNs (2.7 ± 0.21 msec, *P* = 0.0056, Fig. 4D). In contrast, decay times (D1 SPNs = 19 ± 1.7 pA, D2 SPNs = 19 ± 2.0 pA *P* = 0.9524) did not differ between D1 and D2 SPNs (Fig. 4C and E).

### Dendritic architecture

**Figure 5:**
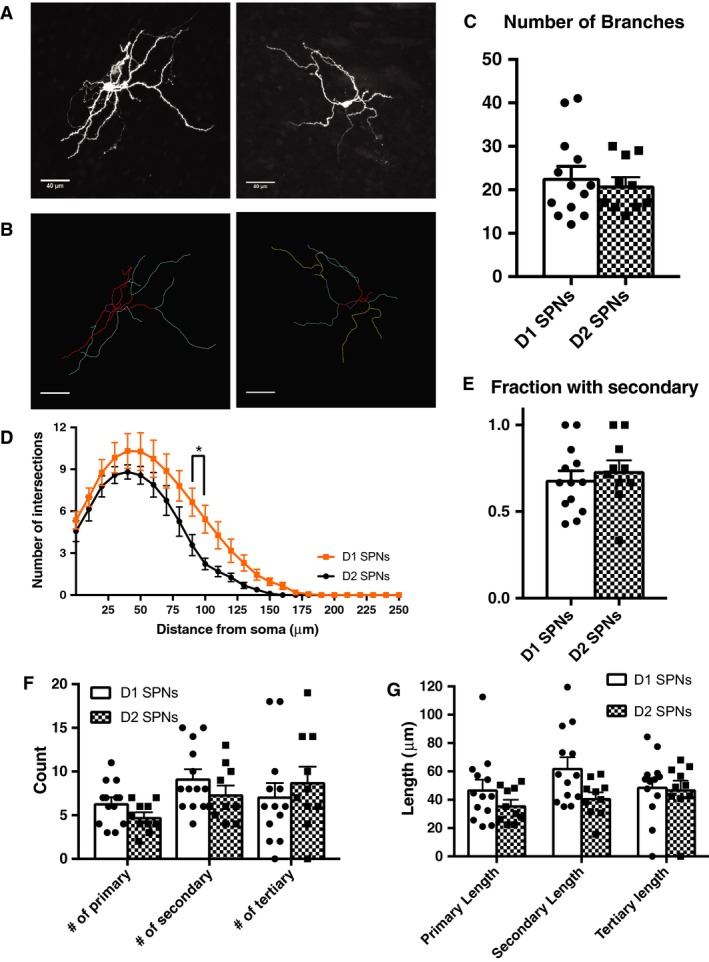
D1 SPN dendrites display increased complexity compared with D2 SPNs. (A) Z projections of confocal images stacks of biocytin filled D1 SPNs (left) and D2 SPNs (right). Slices (250 *μ*m) were stained with fluorescein‐Avidin D. (B) Neurons from Figure 5A traced using the ImageJ plugin NeuronJ. For all analyses, the soma and dendritic arbor were traced. Soma is in magenta, primary dendrites are in red, secondary in cyan, and tertiary in yellow. Images from D1 SPNs (left) and D2 SPNs (right). (C) Total branch number did not differ between D1 and D2 SPNs (*P* = 0.9406). (D) D1 SPN dendrites display increased complexity compared with D2 SPNs. Complexity of D1 and D2 SPNs increased as function of distance from the soma (*F*_25,525_ = 82.75, *P* < 0.0001). There was no significant difference between cell type (*F*_1,21_ = 3.725, *P* = 0.0672), but there was an interaction of these variables (*F*_25,525_ = 1.62, *P* = 0.0301). (E) There was no difference of the fraction of primary branches with secondary branches between D1 and D2 SPNs (*P* = 0.4344). (F) D1 and D2 SPNs did not differ in their number of primary, secondary, or tertiary branches. There was a significant effect of branch complexity on branch number (*F*_2,42_ = 3.952, *P* = 0.0268), but there was no main effect of cell type (*F*_1,21_ = 0.2661, *P* = 0.6114) or a significant cell type by branch complexity interaction (*F*_2,42_ = 1.689, *P* = 0.1971). (G) Branch length did not differ between D1 and D2 SPNs for primary, secondary, or tertiary branches. There was no significant effect of branch complexity on branch length (*F*_2,42_ = 1.538, *P* = 0.2267), or a main effect of cell type (*F*_1,21_ = 4.15, *P* = 0.0544), or a cell type by branch complexity interaction (*F*_2,42_ = 1.384, *P* = 0.2617). *N* = D1 SPNs (13 cells from 10 animals), D2 SPNs (10 cells from 6 animals). Mann–Whitney test was used for total branch count and fraction with secondary. Two‐way ANOVA with multiple comparisons for Sholl analysis, number of individual branches, and branch length.

Alterations of synaptic transmission and excitability have been linked to changes in dendritic complexity (Day et al. 2006; Gertler et al. 2008; Cazorla et al. 2012). Thus, morphological reconstruction was done on biocytin filled neurons, and complexity was assessed using Sholl analysis (Fig. 5A and B). Total branch number did not differ between D1 and D2 SPNs (*P* = 0.9406, Fig. 5C). Sholl analysis revealed that D1 SPNs display a more complex dendritic architecture compared with D2 SPNs (Fig. 5D). Complexity of D1 and D2 SPN dendrites increased as function of distance from the soma (*F*_25,525_ = 82.75, *P* < 0.0001). There was no main effect of cell type (*F*_1,21_ = 3.725, *P* = 0.0672), but there was a distance from soma by cell type interaction (*F*_25,525_ = 1.62, *P* = 0.0301).

The number of primary branches with secondary branches was also assessed (Fig. 5E). D1 and D2 SPNs did not differ in the fraction of primary branches with secondary branches (*P* = 0.4344). Counting of branches by branch type (primary, secondary, tertiary) did not reveal a difference between D1 and D2 SPNs (Fig. 5F). There was a significant effect of branch complexity on branch number (*F*_2,42_ = 3.952, *P* = 0.0268), but no significant difference between cell type (*F*_1,21_ = 0.2661, *P* = 0.6114) or a cell type by branch complexity interaction (*F*_2,42_ = 1.689, *P* = 0.1971).

Branch length did not differ between D1 and D2 SPNs for primary, secondary, or tertiary branches (Fig. 5G). There was no significant effect of branch complexity on branch length (*F*_2,42_ = 1.538, *P* = 0.2267), or a main effect of cell type (*F*_1,21_ = 4.15, *P* = 0.0544), or a cell type by branch complexity interaction (*F*_2,42_ = 1.384, *P* = 0.2617).

## Discussion

Although D1 and D2 SPNs in the dorsal striatum have been well investigated in terms of their differential electrophysiological and anatomical properties, D1 and D2 SPNs of the ventral striatum remain poorly studied (Day et al. 2006; Gertler et al. 2008; Grueter et al. 2010). Through the use of labeled D1 and D2 SPN BAC transgenic mice, we are able to bridge this gap in the literature (Gong et al. 2003; Shuen et al. 2008). While our current studies focused only on neurons that exclusively expressed D1‐ or D2‐type dopamine receptors, there is a population of neurons in the nucleus accumbens core that express both D1‐ and D2‐type dopamine receptors; investigation of this population would thus be of interest. Our present findings complement existing data regarding the properties of D1 and D2 SPNs in the dorsal striatum and provide a more comprehensive characterization of D1 and D2 SPNs in the ventral striatum, in particular the nucleus accumbens core.

Here, we have demonstrated that D1 and D2 SPNs of the nucleus accumbens core exhibit differential electrophysiological properties. D2 SPNs displayed decreased rheobase, increased excitability as measured by action potential firing rates to depolarizing current injections, increased inward rectification, increased input resistance, decreased dendritic complexity, and increased synaptic transmission as compared with D1 SPNs. The two SPN subtypes did not differ in terms of their resting membrane potential, time constant, whole‐cell capacitance, or tonic current.

Passive properties of SPNs differed in the dorsal striatum, where D2 SPNs display decreased capacitance, increased input resistance, and decreased time constant compared with D1 SPNs (Gertler et al. 2008). In the NAcc, we found that capacitance did not differ as a function of cell type. Capacitance is primarily determined by cell size and morphology, consistent with this, in the dorsal striatum D1 SPNs displayed increased dendritic complexity which correlated with their increased capacitance (Gertler et al. 2008). The lack of difference in capacitance between SPN cell types in the NAcc should be interpreted with caution, as the measurement of capacitance via current clamp in complex neurons such as SPNs may underestimate the true value (Golowasch et al. 2009). Input resistance, like capacitance, is partially a function of cell size. In addition, the complement of ion channels expressed by different cells can strongly influence the input resistance. Our results show that D2 SPNs exhibit increased input resistance and Kir ratio concurrent with decreased dendritic complexity. Downregulation of leak channels in D2 SPNs and/or upregulation of leak channels in D1 SPNs may underline the difference in input resistance between the two SPN subtypes. Furthermore, the decreased cell size of D2 SPNs may also contribute to their increased input resistance. This profile is similar to that of the dorsal striatum, where D2 SPNs display both increased input resistance and decreased dendritic complexity (Gertler et al. 2008). Unlike our present findings, dorsal striatal D2 SPNs displayed a decreased time constant, which was driven by their decreased capacitance and cell size (Gertler et al. 2008).

At resting membrane potentials, leak channels and inwardly rectifying K+ channels are the predominant determinants of resting conductance (Uchimura et al. 1989; Nisenbaum and Wilson 1995). SPNs of the nucleus accumbens core did not differ in their resting membrane potential, but there was increased inward rectification (putative Kir) in D2 SPNs compared with D1 SPNs. This difference is the opposite of that reported in dorsal striatum, in which the increased Kir noted in D1 as compared with D2 SPNs was associated with a hyperpolarized resting membrane potential (Gertler et al. 2008). One explanation for the divergent findings between D1 and D2 SPNs in the NAcc may be that leak channels predominate D1 SPNs and I_Kir_ predominates in D2 SPNs. Inwardly rectifying K+ channels are susceptible to changes in membrane potential, whereas leak channels are not. If at rest, resting membrane potential is dictated by leak channels in D1 SPNs and inwardly rectifying K+ channels in D2 SPNs, changes in voltage are more likely to affect D2 SPNs. This would be consistent with the increased input resistance and inward rectification we observed in D2 SPNs. Our findings of decreased rheobase and increased excitability of D2 SPNs are consistent with this interpretation, and likewise similar to the profile reported for SPNs in the dorsal striatum (Gertler et al. 2008).

In addition to potassium channel conductance, several mechanisms control neuronal excitability including cell size and dendritic morphology and receptor expression (Day et al. 2006; Gertler et al. 2008; Cazorla et al. 2012). D1 SPNs displayed increased dendritic complexity compared with D2 SPNs, which is consistent with their decreased excitability. Further mechanisms may also contribute to the differential excitability. In the dorsal striatum, muscarinic M_1_ receptors differentially modulate Kir2 channels in D1 and D2 SPNs. Activation of muscarinic M_1_ receptors inhibits *I*_Kir_ and increases D2 SPN membrane excitability but cannot completely explain the differences in excitability between D1 and D2 SPNs (Shen et al. 2007; Gertler et al. 2008). Moreover, several potassium and calcium channels dictate the excitability of SPNs. For example, work has implicated Kv1.2‐containing K+ channels in the subthreshold excitability of SPNs in the dorsal striatum (Shen et al. 2004). Similar mechanism may exist in the nucleus accumbens core, thus contributing to differential excitability of D1 and D2 SPNs in this region.

In addition to the decreased rheobase, Kir ratio, and increased excitability seen in D2 SPNs as compared to D1 SPNs of the nucleus accumbens core, we also detected changes in synaptic transmission between these cell groups. D2 SPNs displayed a higher frequency of both excitatory (mEPSC) and inhibitory (sIPSCs, mIPSCs) synaptic events as compared with D1 SPNs. The increased mEPSC frequency we found is consistent with a prior report in the accumbens core (Grueter et al. 2010). By contrast, in the dorsal striatum, there does not appear to be a difference in frequency of IPSCs between D1 and D2 SPNs (Ade et al. 2008; Janssen et al. 2009). Our data revealed smaller peak amplitudes and longer rise times of sIPSCs, mIPSCs, and mEPSCs in D2 SPNs. This is consistent with currents deriving from a dendritic source, for example, SPN‐SPN collaterals or a subset of interneurons, suggesting differential synaptic inputs to SPN subtypes (Gittis et al. 2010; Ibanez‐Sandoval et al. 2010; Ünal et al. 2011). In contrast, decay time was faster for mIPSCs in D2 SPNs compared with D1 SPNs. This may suggest an increased proportion of *α*1 containing GABA_A_ receptors at these synapses, as *α*1 containing GABA receptors display faster decay kinetics than *α*2 containing GABA_A_ receptors (Ortinski et al. 2006). In dorsal striatum, IPSC kinetics and amplitude have not been reported to differ between D1 and D2 SPNs (Ade et al. 2008; Janssen et al. 2009). We did not evaluate the subtypes of SPNs either in terms of synaptic inputs or postsynaptic receptor subunit composition; however, differential profiles have been previously reported in dorsal striatum and may influence rise and decay times; the degree to which this holds true in ventral striatum remains to be examined.

Unlike the well‐established difference in tonic current observed between D1 and D2 SPNs of the dorsal striatum (Ade et al. 2008; Santhakumar et al. 2010), we failed to detect a difference in tonic current magnitude between D1 and D2 SPNs in the NAcc. We cannot rule out that TTX may have masked a portion of the BMR‐sensitive tonic current, as these drugs were applied sequentially in this study and independently in prior studies (Ade et al. 2008). Interestingly, subpopulations of both D1 and D2 SPNs displayed both TTX and BMR‐sensitive tonic current. It is plausible that these subpopulations within each SPN type may differ in expression of receptors and/or in their presynaptic partners regulating neurotransmitter release. It remains to be established how ambient GABA and/or glutamate may contribute to the TTX‐sensitive tonic current. In the dorsal striatum, one source of ambient GABA that mediates tonic conductance in SPNs is astrocytic (Wójtowicz et al. 2013). Furthermore, the source of neurotransmitters contributing to the tonic current may be both action potential dependent and independent, perhaps astrocytically mediated (Pandit et al. 2015). In the dorsal striatum, tonic GABA currents are modulated by dopamine receptor signaling and mediated by *β*3 containing GABA‐A receptors. Whether this profile holds true in the accumbens core remains to be examined (Janssen et al. 2011).

In summary, our results demonstrate that there is a physiological dichotomy between D1 and D2 SPNs of the nucleus accumbens core. These differences may, in part, be due to differential channel composition in D1 and D2 SPNs. While differential intrinsic membrane properties, excitability, and synaptic transmission have been reported in dorsal striatal SPN populations, the profile of divergence is different than that observed in the nucleus accumbens core. This suggests that while the dorsal and ventral striatum share a similar basic architecture, there are differences between these regions at the level of cellular neurophysiology. These data provide us with a better understanding of the neuronal circuitry of the ventral striatum and may inform future studies regarding its role in reward and motor function.

## Conflict of Interest

The authors declare no competing financial interests.
